# Multi-Physics Coupling of Rectangular Channels with Different Aspect Ratios in Solid Oxide Electrolysis Cells

**DOI:** 10.3390/ma18122827

**Published:** 2025-06-16

**Authors:** Jie Yao, Carsten Korte, Zhengyang Qian, Ming Chen, Jiangshui Luo

**Affiliations:** 1 Laboratory of Electrolytes and Phase Change Materials, College of Materials Science and Engineering & Engineering Research Center of Alternative Energy Materials & Devices, Ministry of Education, Sichuan University, Chengdu 610065, China; 2 Dongfang Electric (Chengdu) Hydrogen Energy Technology Co., Ltd., Chengdu 611731, China; 3 Institute of Energy Technologies—Electrochemical Process Engineering (IET-4), Forschungszentrum Jülich GmbH, 52425 Jülich, Germany; 4 Zhejiang Youshan New Energy Technology Co., Ltd., Jiaxing 314500, China

**Keywords:** solid oxide electrolysis cell, aspect ratio, multi physical field coupling, three-dimensional model

## Abstract

To explore the impact of the aspect ratio of the channels in the flow fields of solid oxide electrolysis cells on the performance of the cell, we developed three-dimensional models for cells with varying aspect ratios. Our findings revealed that channels with low and high aspect ratios exhibit higher maximum pressure drops, whereas those with medium aspect ratios have the lowest pressure drops. Additionally, the mole fraction of the hydrogen decreases as the channel’s aspect ratio increases. We also computed the polarization curves for SOEC operating under three distinct aspect ratio channels. Our results suggest that structures with low aspect ratios exhibit the poorest electrochemical performance, suitable only for brief operations at low current densities; medium aspect ratio structures exhibit a balanced performance, making them suitable for various operating conditions; and high aspect ratio structures are best suited for operations at high current densities. This study on selecting different aspect ratios aids in determining the optimal channel parameters for different operating conditions, ultimately enhancing the performance of solid oxide electrolysis cells.

## 1. Introduction

Solid oxide electrolysis cells (SOECs) represent a class of high-temperature electrochemical energy conversion devices, that efficiently transform electrical energy into chemical energy through electrochemical processes. These systems enable the conversion of steam or carbon dioxide into hydrogen, carbon monoxide, or syngas via electrochemical reactions. Due to their all-solid-state architecture and elevated operating temperatures (typically 700–1000 °C), SOECs demonstrate exceptional potential in renewable energy storage, carbon cycle utilization, and grid-scale integration of intermittent renewable power sources [1,2].

Structurally, an SOEC comprises three essential components: a fuel electrode (cathode), an oxygen electrode (anode), and a solid oxide electrolyte layer sandwiched between them. The cathode, conventionally fabricated from porous ceramic-metal composites such as nickel–yttria-stabilized zirconia (Ni–YSZ), functions as the active site for steam and CO_2_ reduction reactions. This material combination has been most extensively utilized in SOEC applications due to its balanced electronic/ionic conductivity and remarkable catalytic activity for reduction processes [3]. The anode typically employs perovskite-type oxides, exemplified by lanthanum strontium cobalt ferrite (LSCF), which exhibits superior electrochemical activity, minimized polarization resistance, and excellent operational stability under oxidizing conditions [4]. The intermediate electrolyte layer, predominantly composed of yttria-stabilized zirconia (YSZ), demonstrates exceptional oxygen-ion conductivity at elevated temperatures while maintaining negligible electronic conductivity, thereby ensuring efficient electrolysis operation [5].

**Figure 1:**
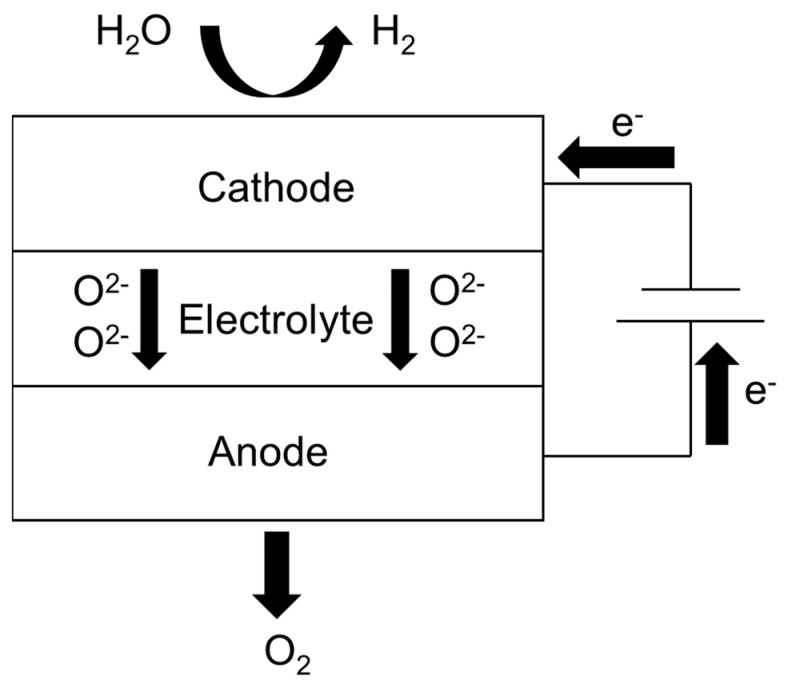
Schematic diagram of the SOEC electrolysis of water.

During operation, a direct current drives the electrochemical dissociation of steam supplied to the cathode side. At the triple-phase boundaries of the Ni–YSZ cathode, water molecules undergo reduction to produce hydrogen gas while releasing oxygen ions. These oxygen ions migrate through the dense YSZ electrolyte layer under the influence of both electrical potential and concentration gradients. Subsequently, the transported ions are oxidized at the LSCF anode, forming oxygen molecules through electron transfer processes [6]. This energy conversion mechanism effectively stores electrical energy in the form of chemical bonds within the generated fuel gases. The schematic representation below illustrates the operational principle of steam electrolysis in SOEC systems, as shown in Figure 1.

On the cathode side, the reaction expression is: 2H_2_O + 4e^−^ → 2H_2_ + 2O^2−^

On the anode side, the reaction expression is: 2O^2−^ → O_2_ + 4e^−^

The high efficiency of SOECs is intricately linked to their high-temperature operating characteristics. High temperatures not only increase the reaction rates of electrolytic reactions with a certain activation energy, reducing the electricity consumption, but also enhance overall energy utilization efficiency through waste heat recovery [7,8]. Furthermore, SOECs exhibit remarkable adaptability to raw materials, enabling them to produce high-purity hydrogen gas through the electrolysis of water vapor alone, convert carbon dioxide into carbon monoxide, and even directly synthesize a mixture of hydrogen and carbon monoxide (synthesis gas) by co-electrolyzing water and carbon dioxide [9,10]. This versatility endows it with unique value in chemical raw material production, carbon capture, and resource utilization. For instance, it can utilize carbon dioxide and waste heat emitted from steel plants to generate synthesis gas, which can then be used to synthesize liquid hydrocarbons or small molecule alcohols [11,12], completing an industrial carbon cycle loop.

**Figure 2:**
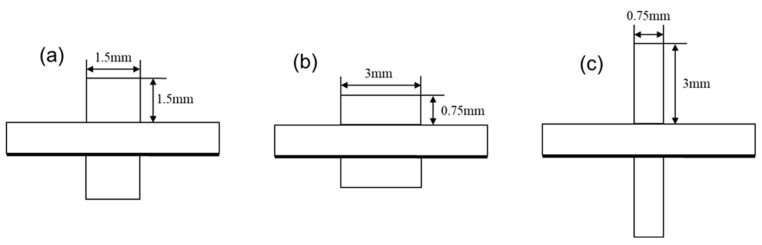
Different aspect ratios of the flow channels: (**a**) aspect ratio = 1, (**b**) aspect ratio = 4, and (**c**) aspect ratio = 0.25.

In solid oxide electrolysis cells (SOECs), the design of the flow channels plays a crucial role in determining the overall performance. Common flow channel designs include parallel, serpentine, and cross-flow configurations. For typical parallel flow channels, both the shape and aspect ratio of the flow cross-section significantly impact the performance of the SOEC. The design of flow channels in SOECs has garnered significant attention in recent years as a crucial aspect of performance optimization. The channel structure directly impacts the distribution of reaction gases, temperature uniformity, and the utilization of electrochemical active areas, thereby determining electrolysis efficiency and long-term stability [13,14]. Current research primarily concentrates on optimizing the geometry of these flow channels, and various studies have been conducted to explore the effects of flow fields on the performance of SOECs and solid oxide fuel cells (SOFCs) using two-dimensional or three-dimensional simulation models [15]. Zhang et al drew inspiration from tree leaves and designed a biomimetic clover-shaped flow field, significantly enhancing the uniformity of battery temperature [15]. Xu et al. crafted SOEC channels of varying cross-sectional shapes, exploring their impact on SOEC performance [16]. In our study, we kept the cross-sectional area of the channels constant while varying the aspect ratio by adjusting the width and height of the cross-section. This approach allows us to investigate the influence of aspect ratio on the performance of a single-channel SOEC. This study developed and examined three-dimensional cell models featuring flow channels of three distinct aspect ratios (Figure 2), comparing and analyzing their performance disparities. As shown in Figure 2, variant (b) offers a wider channel, which increases the contact area between the gas and the electrode surface. This is expected to enhance the overall reaction performance, making this design more promising. These physical considerations provide useful a priori expectations that will guide the interpretation of the subsequent simulation results.

## 2. Model Establishment

### 2.1. Geometric Model

**Figure 3:**
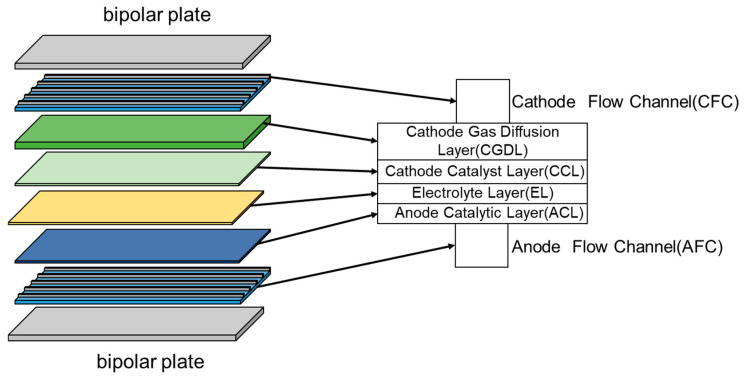
Structure of the SOEC.

Figure 3 shows the detailed structure of the SOEC battery cells, which were designed based on the experimental scheme proposed by Nagata et al. [17]. Among them, the bipolar plate is composed of stainless steel, the cathode gas diffusion layer (CGDL) and cathode catalytic layer (CCL) are composed of Ni–YSZ, the electrolyte layer (EL) is composed of Y_2_O_3_ stabilized ZrO_2_ (YSZ), and the anode catalytic layer (ACL) is composed of LSM.

The detailed geometric dimensions are listed in Table 1.

### 2.2. Mathematical Model

#### 2.2.1. Electrochemical Model

During the electrolysis process, the operating voltage of the SOEC is composed of the equilibrium potential (*E_Nernst_*), an ohmic overpotential (*η_ohm_*), activation overpotential (*η_act_*), and concentration overpotential (*η_conc_*), as shown in Equation (1).

(1) $$V=E_{Nernst}+\eta_{ohm}+\eta_{act}+\eta_{conc}$$

*E_Nernst_* is the equilibrium potential, whose value varies with temperature and pressure, and can be calculated by Equations (2) and (3).

(2) $$E_{Nernst}=E_{Nernst}\left(T\right)+\frac{RT}{nF}ln\frac{P_{O_{2}}^{0.5}P_{H_{2}}}{P_{H_{2}O}}$$

(3) $$E_{Nernst}\left(T\right)=1.229-0.9\times 10^{-3}(T-298.15)$$

In the equation, *R* is the universal gas constant, *T* is the temperature, *n* is the number of electrons transferred in the reaction, and *F* is the Faraday constant. *p_x_* represents (where *x* represents each component) the partial pressure of each component.

The ohmic overpotential is a voltage drop caused by the resistance inside the electrolytic cell or the contact resistance between the interfaces of each component. It follows Ohm’s law and is proportional to the intensity of the current. In SOECs, due to the very low conductivity of the electrolyte, it can be considered that the Ohmic overpotential is mainly caused by the electrolyte [18]. Ohmic overpotential can be calculated using Equation (4).

(4) $$\eta_{ohm}=2.99\times 10^{-5}JL_{EL}exp(\frac{10300}{T})$$

where *J* is the current density and *L_EL_* is the thickness of the electrolyte.

The activation overpotential is the voltage loss caused by the limitations of the electrochemical reaction kinetics in an electrolytic cell. It is an important component of the total overpotential of the electrolytic cell, reflecting the ease of electrochemical reactions on the electrode surface. The activation overpotential can be calculated using the Butler–Volmer equation (Equation (5)) [19], as follows:

(5) $$J=J_{0}[\exp\left(\frac{\alpha nF\eta_{act}}{RT}\right)-\exp\left(-\frac{(1-\alpha )nF\eta_{act}}{RT}\right)]$$

where *J*_0_ denotes the exchange current density and *α* stands for the transfer coefficient, typically set at 0.5.

The concentration overpotential of the electrolytic cell is caused by the difference between the concentration of reactants or products on the electrode surface and the concentration of the solution itself during the electrolysis process. The concentration overpotential is calculated according to Equation (6).

(6) $$\eta_{conc}=\frac{RT}{nF}ln(\frac{P_{bulk}}{P_{sur}})$$

where *p_bulk_* is the bulk concentration of the gas and *p_sur_* is the concentration on the electrode surface.

In the process of SOEC electrolysis, it satisfies the charge conservation equation, as shown in Equations (7) and (8).

(7) $$\nabla \cdot \left(-\sigma_{e}^{eff}\nabla \varphi_{s}\right)=S_{ele}$$

(8) $$\nabla \cdot \left(-\sigma_{i}^{eff}\nabla \varphi_{i}\right)=S_{ion}$$

In the formula, ∇· is the divergence operator, representing the divergence distribution of the vector field and ∇ is the gradient operator, representing the rate and direction of change of the scalar field. The electric potential gradients of electrons and ions are denoted by ∇*φ_s_* and ∇*φ_i_*, respectively. And the source terms of electrons and ions are denoted by *S_ele_* and *S_ion_*, respectively, representing the rate of generation or consumption of electrons or ions per unit volume. The source term of the charge conservation equation can be obtained through the Butler–Volmer equation [20].

#### 2.2.2. Conservation of Mass

During the operation of the electrolytic cell, the transfer of mass units is controlled by the conservation of mass equation. For multi-component mixtures, the Maxwell–Stefan equation describes the interactions between one component and other components. Therefore, we coupled the mass conservation equation with the Maxwell–Stefan equation to describe the transport between components in the electrolytic cell, as shown in Equation (9) [21].

(9) $$\frac{\partial (\varepsilon \rho \omega_{i})}{\partial t}+\nabla \cdot \left(\varepsilon \rho \omega_{i}\right)=-\nabla \cdot j_{i}+S_{i}$$

where *ε* is the porosity, *ρ* is the density, *ω*_i_ is the mass fraction, *j*_i_ is the diffusional mass flux, and *S*_i_ is the mass source term. The mass flux *j*_i_ can be calculated by Equation (10), where the expressions of *S*_i_ are listed in Table 2.

(10) $$j_{i}=\rho D_{ij}^{eff}\nabla \cdot \omega_{i}$$

In porous electrodes, if the pore ratio of the porous electrode is significantly smaller than the average free path of molecules, the diffusion of molecules will still be influenced by the surface walls [22]. In this case, it is necessary to correct the diffusion coefficient of binary molecules (Equation (11)).

(11) $$D_{ij}^{eff}=\frac{\varepsilon }{\tau }D_{ij}$$

where τ is the tortuosity, and the expression for $D_{ij}$ is given by Equation (12).

(12) $$D_{ij}=2.198\frac{T^{1.75}}{P\left({v_{i}}^{1/3}+{v_{j}}^{1/3}\right)}{\left(\frac{1}{M_{i}}+\frac{1}{M_{j}}\right)}^{1/2}$$

#### 2.2.3. Conservation of Momentum

The momentum transfer serves as the foundation for mass transfer during the operation of the electrolytic cell. This process predominantly takes place within the flow channels and porous media regions of the cell. The momentum transfer process in the flow channels can be accurately described by using the Navier–Stokes equation (Equation (13)) [23], while in the realm of porous media, the momentum transfer can be effectively characterized by the Brinkman equation (Equation (14)) [24].

(13) $$\rho \frac{\partial \vec{u}}{\partial t}+\rho \partial \vec{u}\cdot \nabla \vec{u}=\nabla \cdot \left[-pI+\mu \left(\nabla \vec{u}+{\left(\nabla \vec{u}\right)}^{T}\right)-\frac{2\mu }{3}\left(\nabla \cdot \vec{u}\right)I\right]$$

(14) $$\frac{\rho }{\varepsilon }\left[\frac{\partial \vec{u}}{\partial t}+\left(\vec{u}\cdot \nabla \vec{u}\right)\frac{\vec{u}}{\varepsilon }\right]=\nabla \cdot \left[-pI+\frac{\vec{u}}{\varepsilon }\left(\nabla \vec{u}+{\left(\nabla \vec{u}\right)}^{T}\right)-\frac{2\mu }{3}\left(\nabla \cdot \vec{u}\right)I\right]-\left(\frac{\mu }{\kappa }+\frac{S_{m}}{\varepsilon^{2}}\right)\vec{u}$$

where *μ* is the dynamic viscosity of the fluid and *κ* is the permeability of the porous medium material. We use the finite element method (FEM) to discretize the momentum equation and solve the nonlinear equations by Newton’s method to ensure the accuracy and stability of the solution. The physical parameters are listed in Table 3.

#### 2.2.4. Heat Transport

Since the electrodes of SOECs are all porous electrodes, we use the heat conduction convection equation in porous media to describe the heat transfer process of fluids in porous media. It combines the effects of heat conduction, convection, and heat sources, and considers the porosity of porous media, as shown in Equation (15) [15].

(15) $$\frac{\partial }{\partial t}\left(\varepsilon \rho C_{p}T\right)+\nabla \cdot \left(\varepsilon \rho C_{p}\vec{u}T\right)+\nabla \cdot \left(-\lambda \nabla T\right)=Q$$

where *C_p_* is the specific heat capacity of the fluid, *λ* is the effective thermal conductivity of the porous medium, and *Q* is the heat source term, representing the generation or absorption of heat per unit volume. In the electrolysis reaction, *Q* is mainly composed of *Q_J_* generated due to charge transfer, *Q_e_* generated by the electrochemical reaction of electrolyzed water, and *Q_r_* generated by the chemical reactions [15].

### 2.3. Boundary Conditions

Table 4 summarizes the boundary conditions of the SOEC model developed in this study. The inlet temperatures of the cathode and anode of the SOEC are both 1073 K, and the reaction gases are a mixture of ideal gases. The gas composition ratio at the cathode inlet is H_2_O:H_2_ = 6:4, where the gas composition ratio at the anode inlet is N_2_:O_2_ = 0.79:0.21.

### 2.4. Model Validation

**Figure 4:**
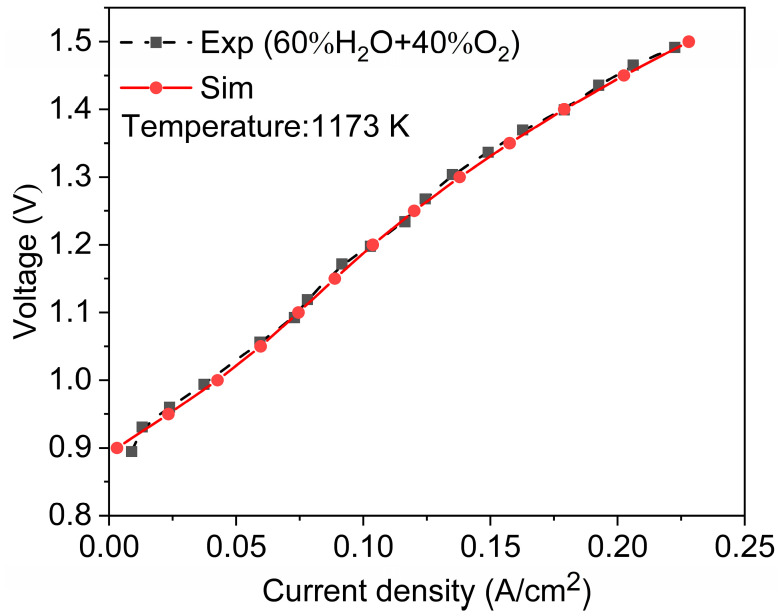
Model validation with experiment data [17].

To verify the accuracy of the model, we compared experimental data and simulated data from the publicly available literature [17]. Both adopt the same geometric model and operating conditions and then analyze their polarization curves (Figure 4). After calculation, the maximum relative error is about 2%. The highly similar polarization curves of the two demonstrate the accuracy of the established model.

## 3. Results and Discussion

### 3.1. Temperature Distribution

**Figure 5:**
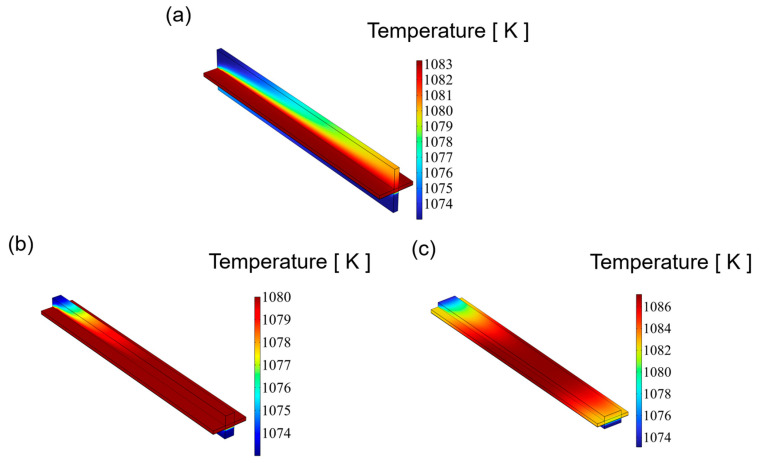
The distribution of temperature for three types of flow channel: (**a**) aspect ratio = 0.25, (**b**) aspect ratio = 1, and (**c**) aspect ratio = 4.

Figure 5 illustrates the temperature distribution of an SOEC at 1.4 V for channels with varying aspect ratios. In multi-physics coupling, the temperature is influenced by the fluid flow. Among three distinct flow fields, the highest temperature is observed on the CGDL, primarily due to the hydrogen electrode as the primary site for electrochemical reactions, which generate significant heat. The highest temperatures, in ascending order of aspect ratio, are 1083.2 K, 1081.6 K, and 1087.1 K. For the SOEC cathode channels with aspect ratios of 0.25 and 1, the temperature distribution is relatively uniform, exhibiting an increasing trend, with the peak temperature occurring at the outlet of the cathode channel. Conversely, for the SOEC cathode channel with an aspect ratio of 4, the highest temperature is found in the middle of the channel, and this maximum temperature surpasses that of the other two channels. Excessive temperature differences can readily induce thermal stress, potentially leading to cracking or even failure of the SOEC. A reduced temperature difference is advantageous for enhancing the lifespan of an SOEC.

### 3.2. Gas Distribution

**Figure 6:**
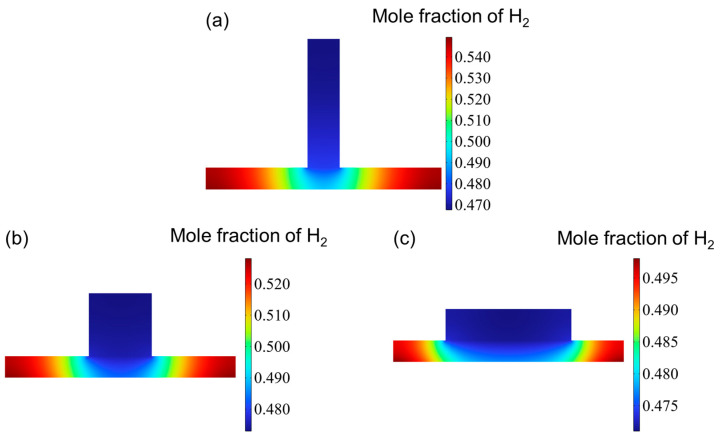
The mole fraction distribution at hydrogen outlet for three types of flow channel: (**a**) aspect ratio = 0.25, (**b**) aspect ratio = 1, and (**c**) aspect ratio = 4.

Figure 6 illustrates the distribution of the hydrogen mole fraction at the outlet of the cathode channel. Overall, the distribution patterns across the three channel configurations are broadly similar. While variations in hydrogen concentration within the channel itself are minimal, more significant differences are observed within the reaction layer. Specifically, the hydrogen mole fraction in the reaction layer exhibits a gradual increase from the center toward both edges, reaching peak values near the channel boundaries. When arranged in order of increasing aspect ratio, the maximum hydrogen mole fractions at the outlet are 0.549, 0.525, and 0.498.

**Figure 7:**
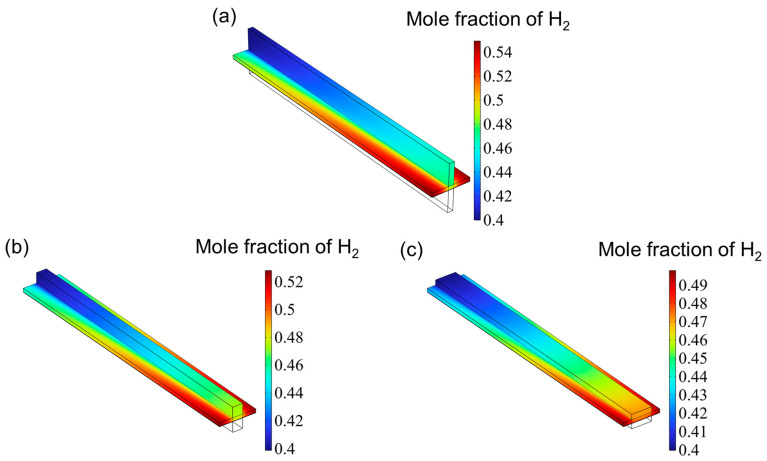
The distribution of hydrogen for three types of flow channel: (**a**) aspect ratio = 0.25, (**b**) aspect ratio = 1, and (**c**) aspect ratio = 4.

**Figure 8:**
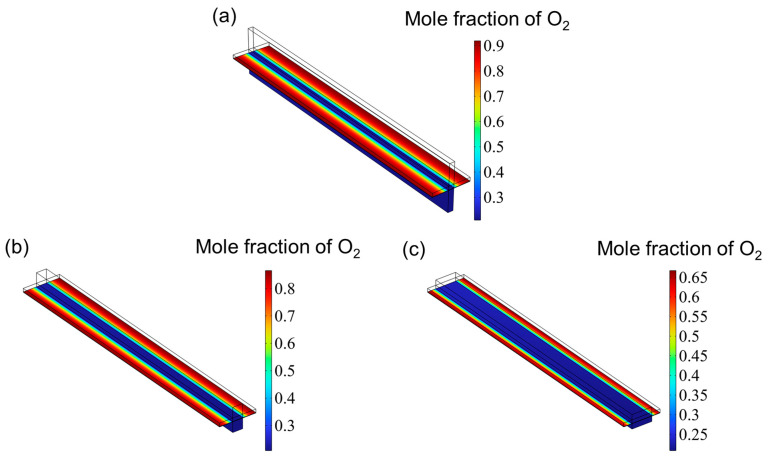
The distribution of oxygen for three types of flow channel: (**a**) aspect ratio = 0.25, (**b**) aspect ratio = 1, and (**c**) aspect ratio = 4.

Figure 7 presents the hydrogen mole fraction distribution across the entire SOEC cathode. The hydrogen concentration increases progressively from the inlet and reaches its peak near the cathode gas diffusion layer (CGDL) at the outlet. The low aspect ratio structure has a higher hydrogen mole fraction because the flow of hydrogen is hindered and cannot be discharged in time, so it accumulates at the interface between the catalyst layer (CL) and the gas diffusion layer (CGDL), forming a local high concentration area. On the contrary, since the high aspect ratio structure has better diffusion characteristics, the generated hydrogen can escape more easily and diffuse into the air. Therefore, the high aspect ratio flow channel has the lowest hydrogen mole fraction. Figure 8 shows the oxygen distribution in the SOEC anode. The overall oxygen profiles for the three flow channel designs are largely similar. Due to the deliberate excess of oxygen supply, the oxygen mole fraction within the anode channel remains relatively constant. In contrast, within the catalyst layer, the oxygen concentration gradually increases from the center toward both edges, reaching a maximum near the rib regions. This is attributed to the generation of oxygen at the triple-phase boundary (TPB), where oxygen ions transported through the electrolyte release electrons to form oxygen gas, which then diffuses into the underlying flow channel. However, the absence of a flow path beneath the ribs hinders timely gas removal, resulting in local accumulation and the formation of high-concentration zones. Similar to the hydrogen distribution in the cathode, the peak oxygen mole fraction in the anode decreases with increasing aspect ratio.

### 3.3. Pressure Distribution

**Figure 9:**
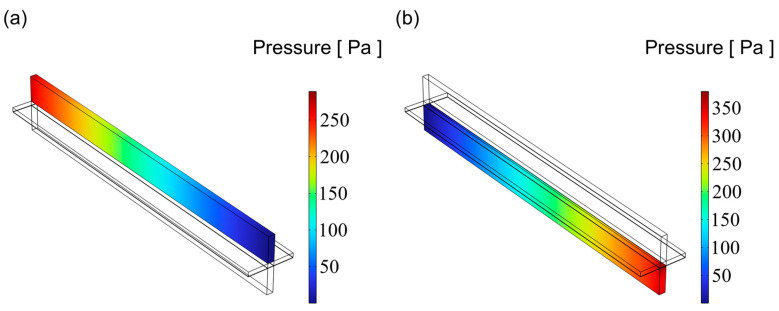
The distribution of pressure for flow channel with an aspect ratio = 0.25: (**a**) cathode channel and (**b**) anode channel.

**Figure 10:**
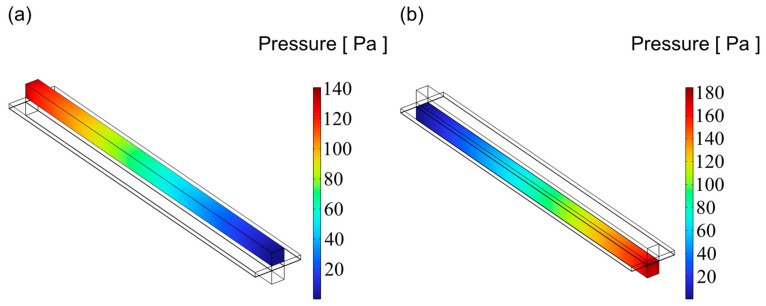
The distribution of pressure for flow channel with an aspect ratio = 1: (**a**) cathode channel and (**b**) anode channel.

**Figure 11:**
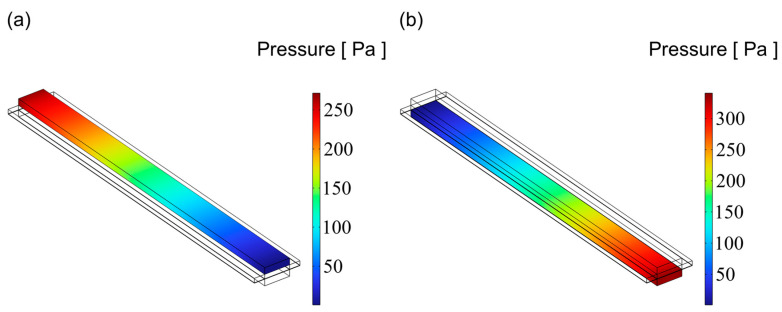
The distribution of pressure for flow channel with an aspect ratio = 4: (**a**) cathode channel and (**b**) anode channel.

Figure 9, Figure 10 and Figure 11 illustrate the pressure distribution within the cathode and anode channels of the SOEC. Excessive internal pressure can induce mechanical stress, potentially leading to crack formation and eventual cell failure. During electrolysis, water vapor in the gas channels is gradually replaced by hydrogen, resulting in a decrease in gas viscosity. Under the condition of a constant inlet cross-sectional area, the pressure drop in a flow channel is primarily influenced by its cross-sectional geometry. When the channel becomes either “narrow and tall” or “wide and flat”, although the cross-sectional area remains unchanged, the surface-to-volume ratio increases significantly. This results in a larger contact area between the gas and the channel walls, thereby enhancing viscous frictional resistance. Consequently, the fluid experiences greater shear forces along the channel, leading to a higher pressure drop. In contrast, a channel with a nearly square cross-section has a smaller wall surface area under the same flow area, resulting in reduced shear stress and thus a lower pressure loss. Additionally, the flow velocity profile within a square channel tends to be more uniform, minimizing velocity gradients between the inlet and outlet, which further contributes to a lower overall pressure drop. Therefore, from the perspective of minimizing flow resistance, a square-shaped channel generally exhibits superior pressure drop performance compared to more elongated geometries. Among the three configurations, the channel with an aspect ratio of 1 exhibits superior flow uniformity, which promotes higher gas flow velocity and consequently results in lower overall pressure values.

**Figure 12:**
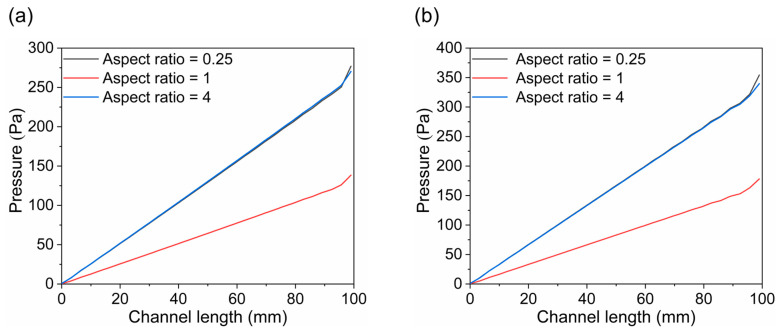
The distribution of pressure along flow channel: (**a**) cathode channel and (**b**) anode channel.

As shown in Figure 12, the pressure distribution trends are generally consistent across all three channel designs, with the anode channel consistently exhibiting higher pressure than the cathode channel. Additionally, the pressure increases progressively along the flow direction in all cases, reaching a maximum at the channel inlet. Notably, the channel with an aspect ratio of 1 shows the lowest pressure across the domain, suggesting a reduced tendency for stress accumulation during operation and, therefore, enhanced structural reliability and service performance.

### 3.4. Cell Performance

**Figure 13:**
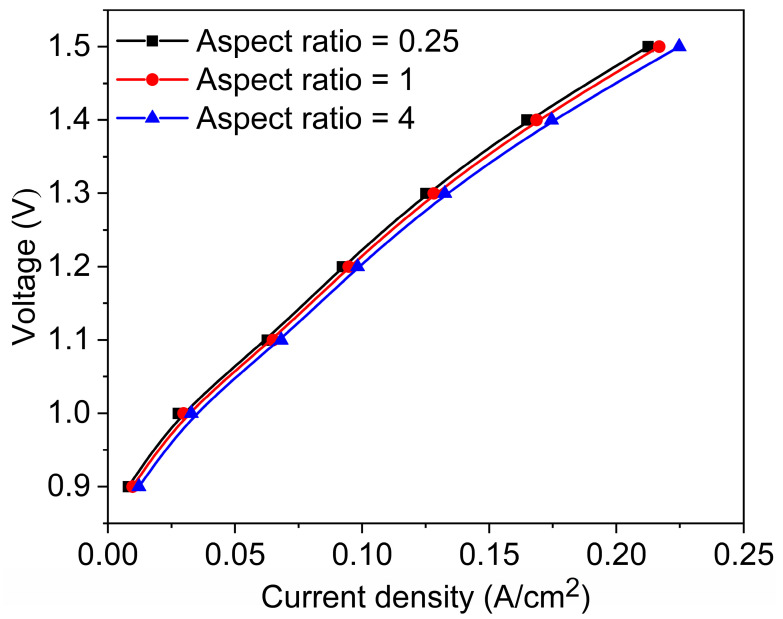
Polarization curves of three types of channels.

Figure 13 illustrates the polarization curves for three channels with different aspect ratios. A lower aspect ratio (aspect ratio = 0.25) corresponds to a narrower and taller channel, which results in a longer gas flow path and reduced flow velocity, meaning the gas requires more time to pass through the entire channel. During this process, the increased surface contact with the channel walls leads to greater frictional resistance, causing a higher pressure drop. This elevated pressure loss contributes to increased ohmic polarization, thereby resulting in a higher overall cell voltage as reflected in the polarization curve. Furthermore, narrow and tall channels often exhibit greater flow non-uniformity, which can lead to uneven gas distribution. Consequently, certain regions of the catalyst layer may be underutilized or suffer from insufficient reactant supply, ultimately reducing the overall reaction efficiency. This design is only suitable for low current density or short-term operations to prevent performance degradation due to gas retention. Conversely, under high aspect ratio conditions (aspect ratio = 4), the channel is wider and shorter, offering reduced gas flow resistance and enhanced mass transfer efficiency. Compared to narrow and tall channels, flat geometries significantly reduce the diffusion path from the bulk flow to the catalyst layer, thereby lowering mass transfer resistance. Moreover, the reduced channel height leads to thinner concentration boundary layers, which enhances the local concentration gradients and accelerates species diffusion. Additionally, flat channels inherently provide a larger surface-to-volume ratio, offering a more extensive gas–solid interface per unit volume and improving the contact efficiency between reactants and the catalyst surface. The shear flow developed along the broader wall surface also helps refresh the boundary layer, promoting convective mixing and further enhancing reactant delivery. As a result, the combination of short diffusion distances, thin boundary layers, and increased interfacial area makes wide and flat channels more effective in sustaining high mass transfer rates, which ultimately contributes to improved electrochemical performance. This configuration can mitigate concentration polarization and enhance electrolysis efficiency. As evident from the graph, the current density range is broader, indicating that a higher current density can be maintained even under high voltage, making it ideal for high current density operations. The balanced design (aspect ratio = 1) not only has good electrochemical performance, but also has simple processing methods and low processing costs. Therefore, it is the simplest flow channel design method, which is widely used in conventional water electrolysis or CO_2_ co-electrolysis.

## 4. Conclusions

In this study, a three-dimensional computational model of a solid oxide electrolysis cell (SOEC) with flow channels of different aspect ratios was systematically developed and validated against experimental data from the published literature [17]. Our analysis shows that the flow channels with low aspect ratio (aspect ratio = 0.25) exhibit the highest hydrogen mole fraction. In contrast, the configuration with high aspect ratio (aspect ratio = 4) increases the contact area of the catalyst layer and reduces concentration polarization while achieving excellent polarization characteristics, making it particularly suitable for high current density operation. The medium aspect ratio (aspect ratio = 1) channels exhibit balanced performance indicators in terms of electrochemical activity, mass transfer efficiency, and pressure drop, which coupled with their simple fabrication process and low manufacturing price, have broad applicability in different operating scenarios. These findings provide important guidance for optimizing the flow channel geometry to meet specific operating requirements in SOEC systems. It is expected that the established framework will facilitate the design of high-performance SOEC architectures suitable for different operating conditions, ultimately promoting the development of efficient energy conversion technologies for renewable hydrogen production.

## Figures and Tables

**Table 1:** Geometric Parameters of the SOEC.

Parameters	Values (m)
CGDL thickness	5 × 10^−4^
CCL thickness	1 × 10^−5^
EL thickness	1 × 10^−5^
ACL thickness	2.5 × 10^−5^
Gas channel width	1.5 × 10^−3^
Gas channel thickness	1.5 × 10^−3^
Gas channel length	9.9 × 10^−2^

**Table 2:** Expressions of *S_i_*_._

Source Term *S_i_*	Expression
*S*_H_2__	$\frac{j}{2F}\times M_{H_{2}}$
*S*_H_2_O_	$-\frac{j}{2F}\times M_{H_{2}O}$
*S*_O_2__	$\frac{j}{4F}\times M_{O_{2}}$

**Table 3:** Physical parameters [25,26].

Parameters	Values
Electrical conductivity (S/m)(CGDL; CCL; ACL)	2.03 × 10^5^–66.09T; 3.27 × 10^6^–1065.3T; 4.2 × 10^7^/T × exp(–1150/T)
Ionic conductivity (S/m) (EL)	3.34 × 10^4^ × exp(–10300/T)
Porosity (CGDL; CCL; ACL)	0.48; 0.335; 0.335
Permeability (CGDL; CCL; ACL)	1 × 10^−13^; 1 × 10^−12^; 1 × 10^−12^
Density(kg/m^−3^) (CGDL; CCL; EL; ACL)	4500; 4500; 8280; 6820
Thermal conductivity (W/(m K)) (CGDL; CCL; EL; ACL)	4; 4; 2; 4
Specific heat capacity(J/(kg K)) (CGDL; CCL; EL; ACL)	431; 431; 600; 470

**Table 4:** Boundary condition parameters.

Parameters	Values
Operating pressure (atm)	1
Operating voltage (V)	1.32
Operating temperature (K)	1073
Inlet gas component of cathode	60 vol% H_2_O, 40 vol% H_2_,
Inlet gas component of anode	79 vol% N_2_, 21 vol% O_2_,

## Data Availability

The original contributions presented in this study are included in the article. Further inquiries can be directed to the corresponding authors.
